# Takotsubo cardiomyopathy after hypoglycemia in a patient with anorexia nervosa

**DOI:** 10.1186/s12991-021-00364-0

**Published:** 2021-09-04

**Authors:** Kota Kikuchi, Norio Yasui-Furukori, Chie Hasegawa, Manami Watahiki, Teruo Inoue, Kazutaka Shimoda

**Affiliations:** 1 Department of Psychiatry, School of Medicine, Dokkyo Medical University, 880 Kitakobayashi, Shimotsuga, Mibu, Tochigi, 321-0293 Japan; 2 Department of Cardiovascular Medicine, School of Medicine, Dokkyo Medical University, 880 Kitakobayashi, Mibu, Tochigi, Japan

**Keywords:** Takotsubo cardiomyopathy, Hypoglycemia, Anorexia nervosa, Eating disorders

## Abstract

**Background:**

Takotsubo cardiomyopathy, also known as “apical ballooning syndrome”, is generally precipitated by endogenous or exogenous stress. Eating disorders are associated with a variety of physical complications.

**Case presentation:**

We present a case involving a 37-year-old Japanese female with anorexia nervosa. She was admitted because of emaciation with shortness of breath and tiredness, and her weight was 30.0 kg (BMI 10.5 kg/m^2^) at this admission. On the afternoon of the first day of hospitalization, a simple measurement caused hypoglycemia (20 mg/dL), and she lost consciousness. On the night of the second day of hospitalization, electrocardiogram showed negative T waves in II, III, aVf, and V1–6. Ultrasound echo showed hypokinesia at the apex of the heart and hypercontraction at the base of the heart. The left ventricular ejection fraction was 20%. Rest and oxygen administration gradually improved her cardiac function; the left ventricular ejection fraction also improved to 50% based on echocardiography. Finally, her weight increased to 43 kg (BMI 15.2 kg/m^2^) with psychiatric treatment, and she was discharged.

**Conclusions:**

The present case shows the clinical features of Takotsubo cardiomyopathy induced by a hypoglycemic event in addition to underlying anorexia nervosa.

## Background

Takotsubo cardiomyopathy, also known as “apical ballooning syndrome”, is a cardiac disease characterized by transient left ventricular apical wall motion abnormality without epicardial coronary artery disease [1]. This cardiomyopathy is generally precipitated by endogenous or exogenous stress, and eating disorders are associated with a variety of physical complications. We report a case of suspected Takotsubo cardiomyopathy after hypoglycemia accompanied by anorexia nervosa.

## Case presentation

**Fig. 1:**
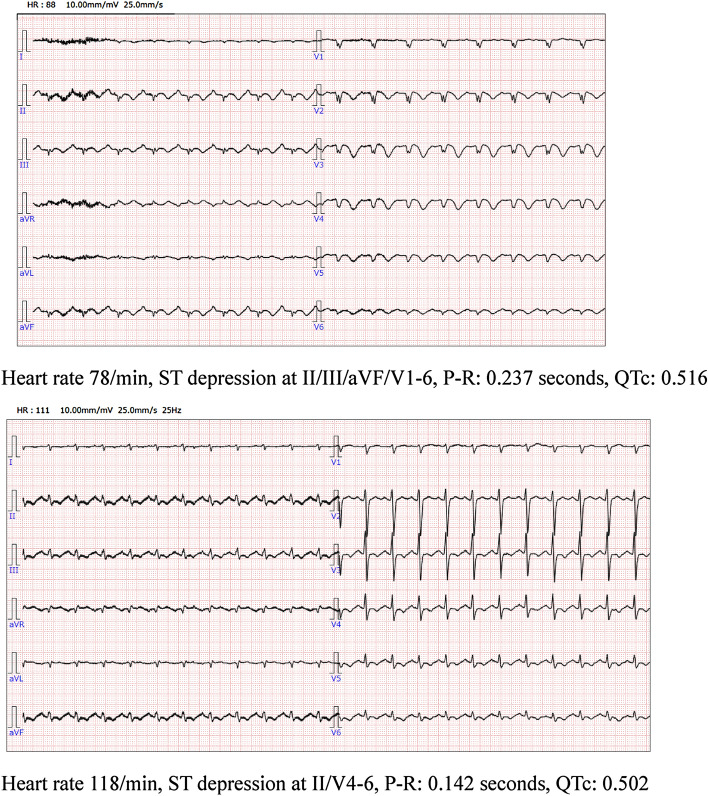
ECGs on the 3rd (upper) and 12th (lower) days of hospitalization

We present a case involving a 37-year-old Japanese female with eating disorders. Eighteen years earlier, she described episodes of binging and purging and weight loss (42 kg; BMI, 16.4) with amenorrhea, intense fear of gaining weight and a distorted view of her body weight and shape. She was diagnosed with anorexia nervosa, binge-purging type according to the DSM-5, and she was admitted to the Department of Psychiatry 5 times because of abnormal weight loss. At the sixth admission, she was admitted because of emaciation with shortness of breath and tiredness, and her weight was 30.0 kg (BMI, 10.5 kg/m^2^). Laboratory tests at admission revealed the following: aspartate aminotransferase, 1731 IU/L; alanine transaminase, 1210 IU/L; gamma-glutamyltransferase, 277 U/L; creatinine, 0.43 mg/dL; urea nitrogen, 29 mg/dL; phosphorus, 3.1 mEq/L; sodium, 137 mEq/L; potassium, 4.5 mEq/L; chlorine, 99 mEq/L; white blood cell count, 2800/µL; red blood cell count, 40.7  ×  109/µL; hemoglobin, 14.1 g/dL; platelets, 9.3  ×  104/µL; and troponin I (sensitive assay), 0.006 ng/mL. Her ECG showed a heart rate of 57 beats/min, P-R interval of 0.247 s, and corrected QT interval of 0.464 s. On the afternoon of the first day of hospitalization, a simple measurement caused hypoglycemia (20 mg/dL), and she lost consciousness. This loss of consciousness was improved by administration of 50% glucose solution, which was coinjected with the maintenance infusion. On the night of the second day of hospitalization, the electrocardiogram monitor revealed tachycardia, and a 12-lead electrocardiogram was performed. Sinus rhythm was observed, but negative T waves were observed in II, III, aVf, and V1–6 (Fig. 1). Ultrasound echo showed hypokinesia at the apex of the heart and hypercontraction at the base of the heart, suggesting Takotsubo cardiomyopathy. The left ventricular ejection fraction was 20%. Refeeding syndrome was also present and became hypophosphatemia, and we administered phosphorus. Rest and oxygen administration gradually improved her cardiac function; the left ventricular ejection fraction also improved to 50% based on echocardiography. Her amount of food intake gradually increased, and we provided psychotherapy. On the 12th day, treatment for eating disorders was continued, as her general condition was improving. Finally, her weight increased to 43 kg (BMI, 15.2 kg/m^2^) with psychiatric treatment, and she was discharged.

## Discussion and conclusions

The present case shows the clinical features of Takotsubo cardiomyopathy induced by a hypoglycemic event in addition to underlying anorexia nervosa. Therefore, patients with severe eating disorders should always be aware of the complications of Takotsubo cardiomyopathy and undergo regular monitoring of blood glucose levels and the cardiovascular system using ultrasound and other methods.

There have been a few case reports of Takotsubo cardiomyopathy and eating disorders [2–11]. In particular, reports of only 5 cases have suggested a relationship between Takotsubo cardiomyopathy and hypoglycemia in addition to eating disorders [3–5, 9, 10]. The average age and range were 29.2 years and 16 and 49 years, respectively, and 4 of 5 patients were female. However, according to epidemiological studies, approximately 90% of Takotsubo syndrome patients are aged 50 years or more [12].

This case meets Takotsubo cardiomyopathy diagnostic criteria I (transient hypokinesis, akinesis, or dyskinesis of the left ventricular midsegments with or without apical involvement; the regional wall motion abnormalities extend beyond a single epicardial vascular distribution; a stressful trigger is often, but not always, present) and III (new electrocardiographic abnormalities (either ST-segment elevation and/or T-wave inversion) or modest elevation in cardiac troponin), but not criteria II (absence of obstructive coronary disease or angiographic evidence of acute plaque rupture) or IV (absence of pheochromocytoma and myocarditis) [13]. Coronary angiography did not rule out a significant lesion, and biopsy did not rule out myocarditis. Therefore, the diagnosis was suspected Takotsubo cardiomyopathy, which was not definitive. Nevertheless, we believe that this case was Takotsubo cardiomyopathy caused by stress due to hypoglycemia.

Although it is difficult to predict Takotsubo cardiomyopathy in advance, frequent measurement of blood glucose levels is necessary to prevent extreme hypoglycemia. In addition, Takotsubo cardiomyopathy commonly occurs in postmenopausal females [12]. One of the risk factors is the decrease in estrogen associated with menopause. In fact, the current patient had experienced irregular menstruation since high school due to anorexia nervosa, and her blood estrogen level was lower (11.8 ng/ml) than that of women of the same age.

In conclusion, the present case shows the clinical features of Takotsubo cardiomyopathy induced by a hypoglycemic event in addition to the underlying anorexia nervosa. Therefore, patients with severe eating disorders should always be aware of the complications of Takotsubo cardiomyopathy and undergo regular monitoring of blood glucose levels and the cardiovascular system using ultrasound and other methods.

## Data Availability

The data used for this case report are available from the corresponding authors upon reasonable request.
